# USING ASSISTIVE TECHNOLOGIES AS A MEASURE TO SUPPORT WELL-BEING IN LATE-LIFE DISABILITIES

**DOI:** 10.1093/geroni/igac059.312

**Published:** 2022-12-20

**Authors:** Tai-Te Su, Shannon Mejía

**Affiliations:** The University of Illinois Urbana-Champaign, Champaign, Illinois, United States; University of Illinois at Urbana-Champaign, Champaign, Illinois, United States

## Abstract

In the United States, the trends of disabilities among older adults have been largely stable over the past decade. However, the impact of disabilities on health and quality of life remains substantial and requires continued research. Along with a set of compensatory strategies, assistive technologies play a promising role in augmenting individuals’ capacity and reducing environmental demands in daily activities. Using data from the five survey rounds of the National Health and Aging Trends Study (2015‒2019), we aimed to investigate the longitudinal associations between disabilities, assistive technologies, and subjective well-being among older adults. A multi-class hierarchical spectrum was constructed to capture the state of disability and assistive technology use. Overall, results showed that subjective well-being decreased progressively along the spectrum. Additionally, assistive technologies were found to differentiate the associations between disabilities and well-being outcomes. Discussions focused on the insights and implications for successful accommodation to disabilities in later life.

